# CASE REPORT How to Repair the Lower Eyelid Retraction, Resulting From the Primary Surgery for Epiblepharon?

**Published:** 2013-10-31

**Authors:** Shinichi Asamura, Hirohiko Kakizaki, Seika Matsushima, Tadaaki Morotomi, Noritaka Isogai

**Affiliations:** ^a^Department of Plastic and Reconstructive Surgery, Kinki University Faculty of Medicine, Japan; ^b^Department of Ophthalmology of the Aichi Medical University, Japan

**Keywords:** epiblepharon, lower eyelid retraction, the posterior layer of the lower eyelid retractors, auricular cartilage, the transcutaneous approach

## Abstract

**Objective:** Among the surgical procedures used to correct the positions of the eyelids or improve their cosmetic appearance, procedures for treating eyelid retraction are some of the most challenging. Lower eyelid retraction can occur iatrogenically after various surgical procedures. We performed a successful corrective procedure for lower eyelid retraction, which had occurred at some point in the 2 decades after primary surgery for epiblepharon. **Method:** A 23-year-old woman underwent primary surgery for bilateral epiblepharon at the age of 5 years. However, at the age of 17, she noticed that an abnormally large proportion of her right sclera was visible when her eyes were in their natural position. In the primary position of gaze, the distance from the lower limbus of the right cornea to the upper margin of the lower eyelid was approximately 2 mm. An incision was made in the lower eyelid along the scar caused by the previous operation. Subsequently, the connections between the tarsus and the lower eyelid retractors were broken, and harvested auricular cartilage was placed between the lower edge of the tarsus and the lower eyelid retractors. **Results:** One year after the operation, there was no gross difference in the distance between the lower margin of the corneal limbus and the lower eyelid on either side, and the patient was extremely happy with the results. **Conclusion:** Using a novel surgical technique, we successfully lengthened the posterior layer of the lower eyelid retractors with a small amount auricular cartilage, resulting in good functional and cosmetic outcomes.

Among the surgical procedures used to correct the positions of the eyelids or improve their cosmetic appearance, procedures for treating eyelid retraction are some of the most challenging. Lower eyelid retraction can be caused iatrogenically by various surgical procedures, including those used to treat epiblepharon.^1^

Thus, when the surgical strategy for reparative surgery for epiblepharon is planned, it is necessary to consider both the functional and cosmetic sequelae. There have been numerous reports about new reparative surgical techniques for epiblepharon and their complications^2^^-^5; however, none of them described the mechanism by which the surgical technique could cause lower eyelid retraction or a detailed explanation of how such complications should be repaired.

We performed a successful corrective procedure for lower eyelid retraction, which occurred during the 2 decades after primary surgery for epiblepharon.

## CASE REPORT

**Figure 1:**
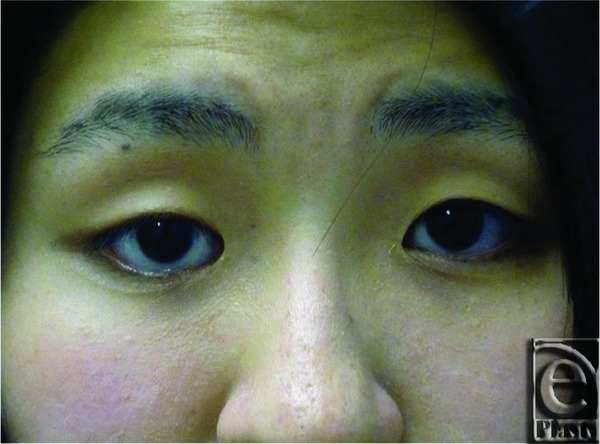
Preoperative view. In the primary position of gaze, the distance from the lower limbus of the right cornea to the upper margin of the eyelid was approximately 2 mm.

A 23-year-old woman underwent primary reparative surgery for bilateral epiblepharon at the age of 5 years. Postoperatively, there were no serious complications in the first postoperative year. However, at the age of 17, she noticed that an abnormally large proportion of her right sclera was visible when her eyes were in their natural position. Five years later, she finally decided to undergo corrective surgery (Fig 1).

The initial physical examination confirmed that apart from her cosmetic eye problem the woman was otherwise healthy. In the primary position of gaze, the distance from the lower limbus of her right cornea to the upper margin of her lower right eyelid was approximately 2 mm, whereas the lower limbus of the patient's left cornea was almost touching the upper margin of the lower left eyelid.

**Figure 2:**
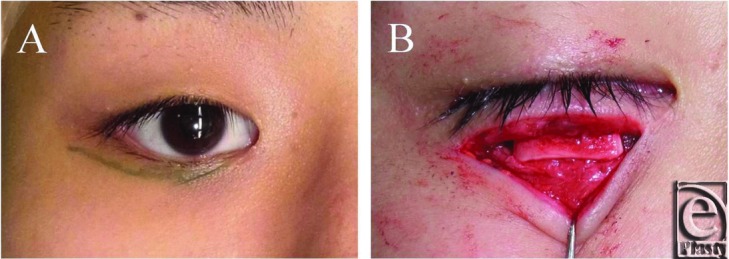
Operative findings. (*a*) The incision in the lower eyelid was made along the scar produced by the previous surgical procedure. (*b*) The harvested auricular cartilage was placed between the lower edge of the tarsus and the posterior layer of the lower eyelid retractors.

To repair the retracted right lower eyelid, first, local anesthesia was induced with 2 mL of 2% lidocaine and epinephrine (1:100,000 dilution). Then, an incision was made in the lower eyelid along the scar caused by the previous surgical procedure (Fig 2a). Although it was technically difficult to separate the lower eyelid retractors from the palpebral conjunctiva, the separation of the anterior and posterior lower eyelid retractors was successfully achieved. During this process, a certain amount of the scar tissue caused by the previous operation was also removed. Subsequently, the connections between the tarsus and the posterior lower eyelid retractors were broken.

Auricular cartilage was harvested via an incision made in the reverse side of the external ear and placed between the lower edge of the tarsus and the posterior layer of the lower eyelid retractors (Fig 2b). The cartilage was fixed to the edge of the tarsus and the posterior layer of the lower eyelid retractors with 6-0 nylon sutures, respectively. The length of the horizontal implanted auricular cartilage was approximately 11 mm, and its vertical height was approximately 3 mm. The anterior layer of the lower eyelid retractors was left intact. At the end of the procedure, the pretarsal orbicularis oculi muscle and the lower edge of the tarsus were secured and permanently enclosed so that they did not touch the cilia on the ocular surface. The skin incision was then sutured with interrupted 6-0 nylon sutures.

**Figure 3:**
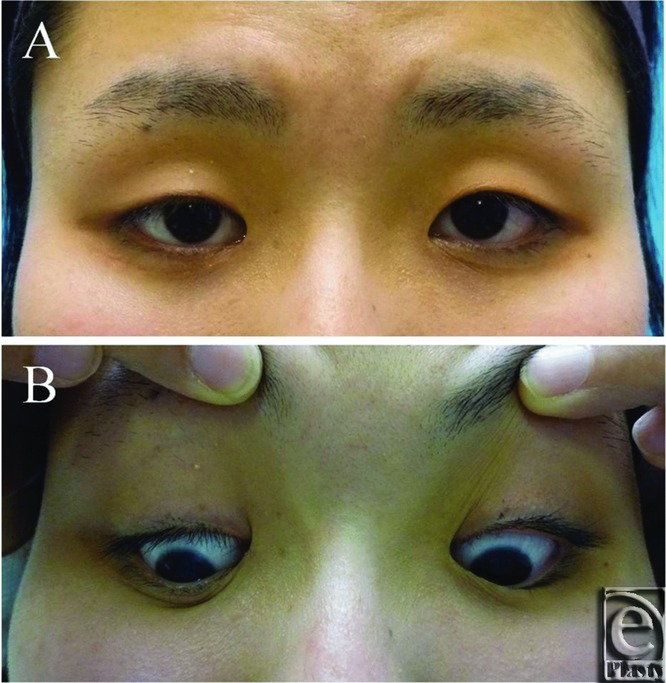
Postoperative view. (*a*) Sufficient elevation of the lower eyelid was achieved. (*b*) The corrected right lower eyelid moved down sufficiently during downward gazing.

The patient did not exhibit exposure keratitis or wound infection during the postoperative period. One year after the repair, there was no gross difference in the distance between the lower margin of the corneal limbus and the lower eyelid on either side. In addition, the corrected right lower eyelid moved down sufficiently during downward gazing, and the patient appeared to be extremely happy with the results (Fig 3).

## DISCUSSION

**Figure 4:**
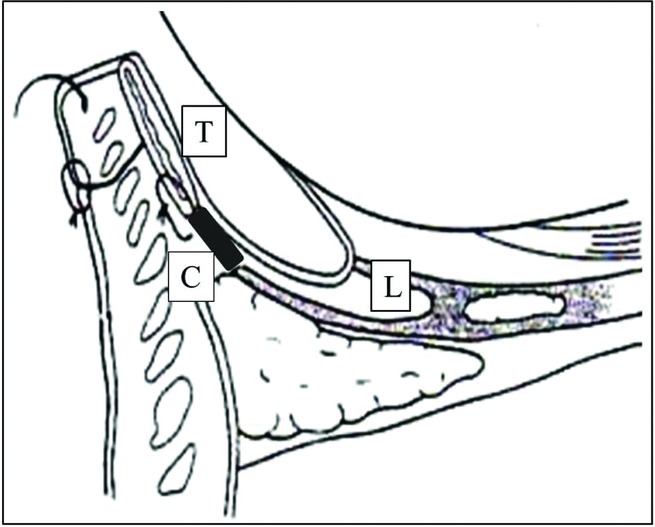
Schematic drawing of our surgical technique. It is important to add auricular cartilage to the defect between the tarsus and the edge of the lower eyelid retractors. T: tarsus, C: cartilage, L: lower eyelid retractors.

If lower eyelid retraction occurs following primary surgery for epiblepharon, it is important to add tissue to the defect between the tarsus and the edge of the posterior layer of the lower eyelid retractors. In the present case, the posterior layer of the lower eyelid retractors was successfully lengthened with a small amount of auricular cartilage, resulting in good functional and cosmetic outcomes (Fig 4).

Lower eyelid retraction results in an abnormal proportion of the scleral tissue between the lower corneal limbus and lower eyelid being exposed when the eye is in its natural position. In general, the most common causes of lower eyelid retraction are thyroid-associated orbitopathy and complications after reparative surgery for strabismus.^1^^,^^6^^,^^7^ In such cases, the posterior lamella of the lower eyelid is shortened due to the contraction of the scar tissue caused by the primary surgery/eye condition. Therefore, theoretically such problems can be fixed by lengthening the posterior lamella.

The transconjunctival approach, in which the posterior lamella is lengthened using mucosal tissue as a spacer, is the most commonly used corrective procedure for lower eyelid retraction.^8^^,^^9^ Hard palate or nasal turbinate mucosal tissue is often used as an autologous spacer since the surfaces of these tissues possess an appropriate degree of rigidity.^10^^-^12 Although keratinization can occur, it only irritates the ocular surface.^9^^,^^12^^,^^13^ As an alternative spacer material, auricular cartilage can be utilized. In such cases, some of the cartilage can remain exposed after the initial procedure and require removal.^9^^,^^14^^,^^15^ In many cases, lengthening the posterior lamella via the transconjunctival approach does not produce good long-term results.

On the contrary, the transcutaneous approach involves the replacement of the entire length of the lower eyelid with auricular cartilage.^16^^,^^17^ Although this results in a good degree of eyelid lifting, it often causes immobility of the lower eyelid during downward gazing.^17^^,^^18^ Cosmetically, the lower eyelid appears to be slightly thicker after cartilage insertion compared with the eyelid on the normal side.^17^^,^^18^ In addition, since a rather large amount of tissue is harvested from the external ear, cosmetic issues are inevitable. However, we suspect that the main cause of cosmetic eyelid problems is excessive surgical manipulation of the posterior lamella, mainly the lower eyelid retractors, resulting in severe scar formation, as the subsequent cicatricial contraction of the surgical wound leads to shortening of the lower eyelid retractors, causing downward retraction of the lower eyelid.

The lower eyelid retractors were originally thought to be composed of a single layer.^19^ However, it was recently demonstrated that they are composed of a double layer, consisting of anterior and posterior layers.^20^ The posterior layer, which is composed of dense capsulopalpebral fascia fibers interspersed with scattered smooth muscle fibers and is attached to the tarsus, is the main pulling structure of the lower eyelid retractors. We consider that scar rigidity and fibrous contracture are the main causes of eyelid retraction after primary eyelid surgery.^21^^,^^22^

Therefore, in our procedure as much as possible of the contracted scar tissue is removed, and then the lower eyelid retractors are visually identified. After the scar tissue in the posterior lamella has been removed, the connections between the tarsus and lower eyelid retractors are broken. Then, the defect between the tarsus and the posterior layer of lower eyelid retractors is filled with harvested auricular cartilage as a spacer. The amount of cartilage used should be sufficient to connect these structures; however, we consider that it is not necessary to fill the defect completely. Cosmetically, the incision used to harvest the cartilage should be made on the reverse side of the ear to minimize its visual impact. The amount of cartilage harvested in the present case was so small that the ear deformity was hardly visible. Regarding the normal position of the lower eyelid, its upper margin should be about 2 mm above the lower corneal limbus in the upright position, and it is also important to consider the effects of gravity and edema during surgery.

Most epiblepharon patients undergo primary surgery during childhood, when their facial bone structures have not fully matured. However, postoperative follow-up is usually only performed for a few years after reparative surgery.^2^^-^5 Accordingly, some epiblepharon patients who undergo primary surgery at a young age subsequently consult specialists because they are dissatisfied with the results of the primary surgery, particularly when they reach puberty. However, we suspect that there is a population of patients who are unsatisfied with the results of their primary surgery but have not consulted a specialist for various reasons.

To diagnose eyelid retraction, thorough knowledge of the normal position of the eyelid and the level of the palpebral fissure is required. Age, direction of gaze, and proptosis can also affect eyelid position. Most cases are treated with primary surgery. However, even if the original surgery is perfect the patient's anatomical structures can change markedly as they get older; therefore, long-term follow-up is necessary to confirm the results of surgery.
